# The first step toward genetic selection for host tolerance to infectious pathogens: obtaining the tolerance phenotype through group estimates

**DOI:** 10.3389/fgene.2012.00265

**Published:** 2012-12-14

**Authors:** Andrea B. Doeschl-Wilson, Beatriz Villanueva, Ilias Kyriazakis

**Affiliations:** ^1^The Roslin Institute and Royal (Dick) School of Veterinary Studies, University of EdinburghEdinburgh, UK; ^2^Departamento de Mejora Genética Animal, INIAMadrid, Spain; ^3^School of Agriculture, Food and Rural Development, Newcastle UniversityNewcastle upon Tyne, UK

**Keywords:** tolerance, resistance, phenotype, infectious disease, livestock, genetic selection

## Abstract

Reliable phenotypes are paramount for meaningful quantification of genetic variation and for estimating individual breeding values on which genetic selection is based. In this paper, we assert that genetic improvement of host tolerance to disease, although desirable, may be first of all handicapped by the ability to obtain unbiased tolerance estimates at a phenotypic level. In contrast to resistance, which can be inferred by appropriate measures of within host pathogen burden, tolerance is more difficult to quantify as it refers to change in performance with respect to changes in pathogen burden. For this reason, tolerance phenotypes have only been specified at the level of a group of individuals, where such phenotypes can be estimated using regression analysis. However, few stsudies have raised the potential bias in these estimates resulting from confounding effects between resistance and tolerance. Using a simulation approach, we demonstrate (i) how these group tolerance estimates depend on within group variation and co-variation in resistance, tolerance, and vigor (performance in a pathogen free environment); and (ii) how tolerance estimates are affected by changes in pathogen virulence over the time course of infection and by the timing of measurements. We found that in order to obtain reliable group tolerance estimates, it is important to account for individual variation in vigor, if present, and that all individuals are at the same stage of infection when measurements are taken. The latter requirement makes estimation of tolerance based on cross-sectional field data challenging, as individuals become infected at different time points and the individual onset of infection is unknown. Repeated individual measurements of within host pathogen burden and performance would not only be valuable for inferring the infection status of individuals in field conditions, but would also provide tolerance estimates that capture the entire time course of infection.

## Introduction

Improvement of host responses to infectious challenges by genetic means is now widely recognized to be a valuable complement to conventional disease control in livestock. Disease traits have been difficult to target by traditional selection, but recent developments in high throughput genomics provide opportunities to dissect host responses to infectious pathogens and to increase the accuracy of selection. Resistance and tolerance are two distinct mechanisms of host response to infectious pathogens that could be targeted for genetic improvement. Resistance refers to the host ability to reduce pathogen invasion or replication, whereas tolerance refers to the host ability to maintain performance and fitness counteracting thus the damage that pathogens can inflict on it. Consequently, resistance is typically described as an inverse measure of pathogen burden (Råberg et al., 2007), whilst tolerance is described in terms of change of host performance or fitness as a result of change in pathogen burden (e.g., Simms, 2000).

Genetic analyses of disease data focus mainly on resistance mechanisms. State-of the art methods in genetic analysis of resistance of livestock to infectious disease have been discussed and outstanding challenges for obtaining reliable estimates of resistance parameters have been highlighted (e.g., Morris, 2007; Bishop and Woolliams, 2010; Ødegård et al., 2011; Bishop et al., 2012). Tolerance mechanisms as a host defense strategy have been extensively studied in plant species (Caldwell et al., 1958; Schafer, 1971). In animals, awareness of the important role of tolerance is rapidly increasing in immunology and evolutionary ecology (Råberg et al., 2007, 2009; Read et al., 2008; Ayres and Schneider, 2012; Medszhitov et al., 2012). However, in the context of livestock breeding, where “breeding for disease resistance” has attracted a significant research effort, it appears that very little attention has been paid to the “breeding for increased tolerance.”

The lack of attention to tolerance of livestock to infectious pathogens in the scientific literature is surprising, given the increasing need to make livestock production more efficient and sustainable in the face of challenges arising from the demands on global food production and climate change (Foresight annual review, 2011). Given that pathogen challenges are ubiquitous and manifold, “maintaining performance in the face of infectious challenge” or “reducing the impact of pathogens on performance” (i.e., the very definition of tolerance), appears to be a valuable breeding goal, at least at first instance. Also, both theoretical and empirical evidence suggest that a trade-off between resistance and tolerance may exist (e.g., Simms and Triplett, 1994; Mauricio et al., 1997; Pilson, 2000). This would imply that attempts to control infectious disease in a population by improving host resistance without considering the consequences on performance may fail if resistance and tolerance are antagonistically related (Doeschl-Wilson et al., 2009a,b).

There are several potential reasons why improvement of host tolerance to pathogens has received relatively little attention in livestock breeding. Some of these reasons are outlined below and constitute the first part of this paper. A close examination of these lead us to hypothesize that genetic improvement of host tolerance to infectious pathogens may be first of all handicapped by our ability to obtain reliable estimates of tolerance at a phenotypic level. Therefore, the aim of this article and its companion paper (Doeschl-Wilson et al., 2012) is to establish what measurements are needed to obtain accurate phenotypic tolerance estimates for genetic studies, and which factors need to be considered in the statistical analyses involved in such studies. Here, generic theoretical concepts for obtaining tolerance phenotypes are presented and their implementations for estimating tolerance for a group of individuals are discussed. In the companion paper we address the question whether tolerance can also be estimated at the level of individuals.

## Why has genetic improvement of tolerance received little attention in livestock genetic research?

There are at least four potential reasons for the apparent scarcity on host tolerance in livestock in genetic research.

### The importance of distinguishing between resistance and tolerance in the animal breeding context has not been brought to attention

The ambiguity and frequent misuse of the terminology when referring to disease traits would support this hypothesis. Whilst “breeding for disease resistance” has become a well-established term, closer inspection reveals that it is not always clear whether the disease trait under consideration refers to resistance rather than to tolerance. For example, infection-induced mortality is a trait commonly used when describing disease resistance in farm species, particularly in fish (Houston et al., 2010; Ødegård et al., 2011). Mortality could actually refer to host resistance, where the animal dies because it cannot control pathogen replication, although the actual damage inflicted by a unit of pathogens may be low. On the other hand, mortality could also refer to tolerance, where the animal dies as a result of much damage inflicted by a unit of pathogens, although the actual pathogen burden may be low.

Another example where resistance and tolerance are frequently confused or used interchangeably, is when dealing with the trypano-tolerance of ruminants, which often refers to disease resistance mechanisms (Naessens, 2006) or is used to encompass both resistance and tolerance traits (Kemp and Teale, 1998). For example, Kemp and Teale (1998) state that “trypano-tolerant cattle show a remarkable *resistance* to the effects of African trypanosomiasis: they can tolerate the presence of parasites while apparently controlling levels of parasitaemia and, crucially not showing the severe anemia and production loss that are characteristics of infection in susceptible hosts.”

Both host resistance and tolerance enhance host fitness, but distinguishing between these mechanisms is critical in genetic improvement programs, not only because they may be to be antagonistically related (Simms and Triplett, 1994; Fineblum and Rausher, 1995; Tiffin, 2000; Blanchet et al., 2010), but also because they can lead to strikingly different epidemiological and evolutionary outcomes, as outlined below.

### Genetic improvement of resistance is considered favorable over genetic improvement of tolerance

Genetic improvement of host resistance as a disease control strategy may be thought favorable over improving tolerance due to their different epidemiological and evolutionary consequences. For instance, disease eradication in a population can only be achieved through increasing resistance, as improving tolerance does not constrain pathogen replication (Roy and Kirchner, 2000). Epidemiological theory further suggests that a threshold density of susceptible hosts is needed for an infection to spread effectively in a population (Keeling and Rohani, 2008). Thus, genetic selection may strive toward generating a sufficiently large proportion of resistant individuals to prevent epidemic outbreaks (MacKenzie and Bishop, 1999). Genetic selection for pathogen resistance has indeed led to reduced disease prevalence in farm species, as exemplified in the case of scrapie in sheep (Baylis et al., 2004), *Escherichia coli* F18 infections in pigs (Meijerink et al., 1997 and intestinal pancreatic necrosis in salmon Ødegård et al., 2011; Houston et al., 2010). However, evolutionary theory suggests that genetic selection for disease resistance may increase pathogen virulence, which should not occur when selecting for tolerance (Roy and Kirchner, 2000). This host-pathogen coevolution may counteract the short-term benefits of genetic selection on animal health, as demonstrated in the case of Mareks disease in poultry (Zelnik, 2003), where selection has only led to short-term reduction in disease prevalence. Indeed, it has been argued that increases in tolerance by selective breeding may be more evolution-proof than manipulations in resistance, because tolerance does not impose selection for pathogen counter-measures (Rausher, 2001).

Genetic improvement of host tolerance may thus be desirable in cases where overall host resistance is low leading thus to high infection prevalence in the population and low chance of elimination of the infection from the population, as is the case for nematode infections or mastitis in ruminants (Bishop, 2012). In fact, a recent simulation study modeling mastitis in dairy cattle (Detilleux, 2011) suggested that under certain conditions increasing individual tolerance could be more effective for maintaining population health and performance than increasing individual resistance. Accumulated theoretical and empirical evidence would thus suggest that it is not *a priori* evident whether selection for host resistance is favorable over selection for host tolerance or *vice versa*. The answer is likely to be case specific and will depend on of both host and pathogen properties.

### Genetic selection for improved host tolerance is not possible due to lack of genetic variation

The existence of genetic variation (heritability) for the trait under consideration is a fundamental requirement for achieving genetic improvement through selection. Evolutionary arguments suggest greater genetic variation in host resistance than in tolerance. For instance, Read et al. (2008) indicated that “the scientific focus on resistance may be because parasite killing mechanisms are more likely to be genetically variable because of host–parasite coevolution.” Furthermore, Roy and Kirchner (2000) argued on theoretical grounds that a tolerance gene should be more likely to be driven to fixation by natural selection than a resistance gene, and supported their theoretical concept with a number of examples across diverse plant species where resistance genes tended to be polymorphic and tolerance genes tended to be fixed. The theory has been supported in animal species; for example, a recent study identified genetic variation in resistance, but not in tolerance of monarch butterflies to a protozoan parasite (Lefèvre et al., 2011).

However, numerous empirical studies in a variety of plant and animal species provide evidence to the contrary (Simms and Triplett, 1994; Fineblum and Rausher, 1995; Mauricio et al., 1997; Koskela et al., 2002; Råberg et al., 2007; Blanchet et al., 2010) and would suggest that genetic variation in tolerance is actually a common phenomenon. For this reason, several theoretical arguments have been put forward to reconcile the apparently contradictory empirical findings with existing theory. These include genetic trade-offs between host fitness in pathogen free environments and tolerance (Agrawal et al., 1999; Tiffin and Rausher, 1999), or tolerance mechanisms acting on fecundity rather than on host survival (Best et al., 2008), as potential mechanisms responsible for maintaining genetic variation in tolerance. These arguments support the existence of genetic variation in host tolerance in animal species.

### Obtaining reliable tolerance phenotypes for genetic analyses is challenging

Resistance and tolerance cannot be measured directly but need to be inferred from more readily available measures of other traits. As resistance refers to mechanisms that reduce pathogen invasion or replication within a host it is typically defined as the inverse of within host pathogen burden (number or mass of parasites per host or per unit host tissue) (Simms and Triplett, 1994; Råberg et al., 2007; Kause, 2011). Tolerance, on the other hand, is defined as the rate of change in host fitness with regards to changes in pathogen burden, and as such is consistent with the definition of the slope when regressing fitness against pathogen burden (Simms and Triplett, 1994; Simms, 2000; Råberg et al., 2009; Kause, 2011).

The concept of tolerance originates from evolutionary ecology and thus the generic term “fitness” has been widely used as response variable for describing tolerance. In animal science, depending on the type of disease, species and breeding goal, the most appropriate choice of response variable may be a fitness related trait (e.g., reproduction or survival trait), but also a measurable production trait. From now on we will use the term performance as a generic term for the response variable when defining tolerance.

The concept of tolerance is simple: a slope value of zero refers to complete tolerance, negative slopes to incomplete tolerance where host performance is reduced due to pathogens, and positive slopes to a mutualistic relationship between host fitness and the pathogens (sometimes called overcompensation). In case of incomplete tolerance, the steeper the slope, the lower the tolerance. However, as outlined in detail below, obtaining accurate phenotypes for this trait is challenging, partly because tolerance refers to a rate of change of a measurable quantity rather than to the quantity itself. We consider the difficulties entailed in estimating tolerance to be the main bottleneck why breeding for tolerance in livestock has received little attention. For this reason specifying the tolerance phenotype, both at theoretical and practical level, constitutes the main focus of our paper.

## Theoretical considerations when specifying the tolerance phenotype

### Specifying pathogen burden

#### The need to measure pathogen burden when quantifying tolerance

Numerous studies have investigated the impact of infection on performance (e.g., Van der Waaij et al., 2000; Vagenas et al., 2007; Doeschl-Wilson et al., 2008, 2009a; Lewis et al., 2009) and compared the performance of animals in non-infectious and infectious environments without quantifying the actual pathogen burden (e.g., Mackinnon et al., 1991; Bisset and Morris, 1996; Naessens, 2006; Doeschl-Wilson et al., 2009b). Do such studies provide useful information on host tolerance?

The ability of animals to maintain relatively undiminished performance levels whilst infected is usually called *resilience* (Albers and Gray, 1986; Bisset and Morris, 1996). Thus resilience and tolerance are both concerned with the impact of infection on performance. However, whereas the definition of tolerance as a rate of change in performance due to changes in pathogen burden implies that tolerance cannot be inferred without quantifying pathogen burden, resilience studies usually do not include a measure of pathogen burden. Instead variation in performance is assessed in relation to an unknown standard level of pathogen challenge to which all individuals are assumed to be equally exposed (Bisset and Morris, 1996). As a consequence of this resilience conflates resistance and tolerance.

**Figure 1:**
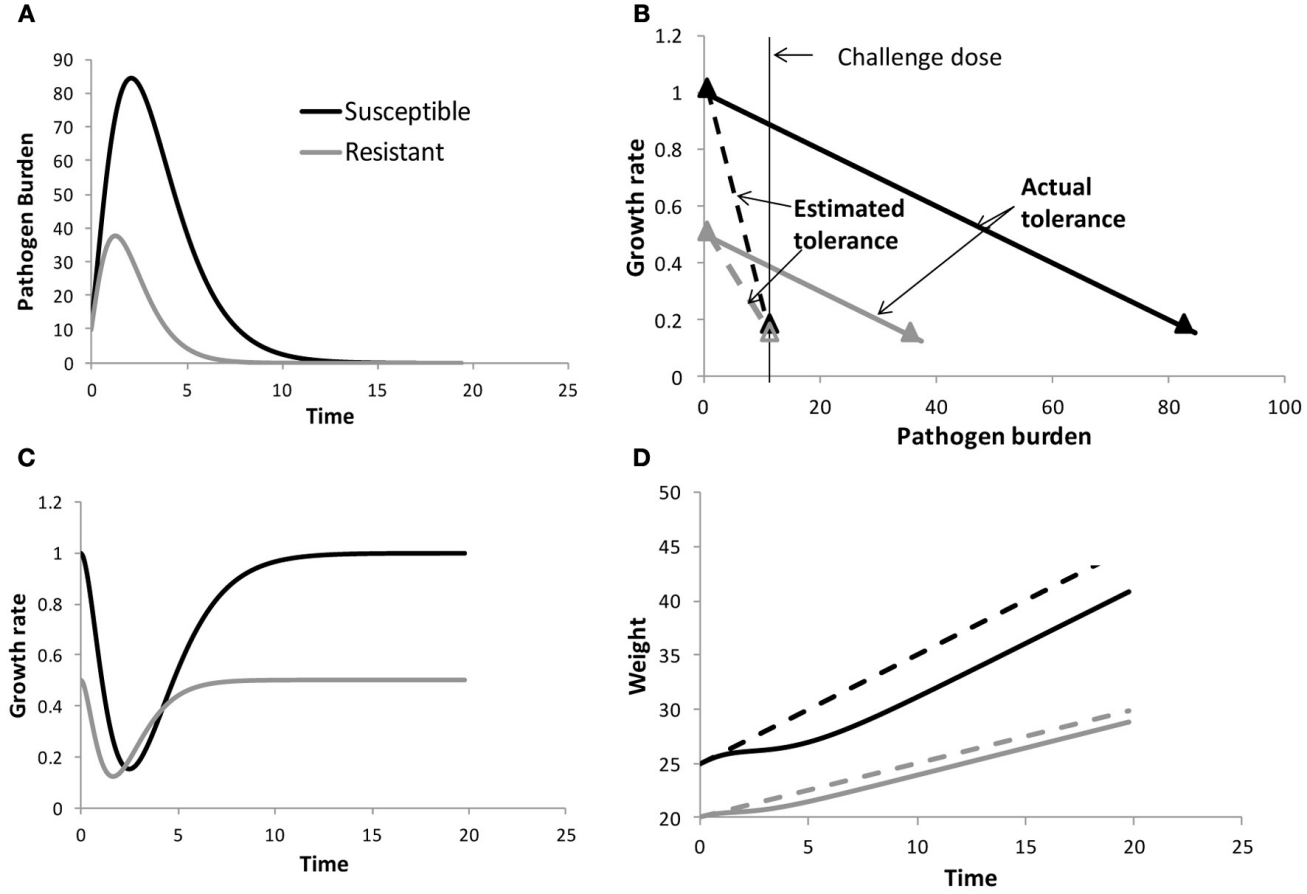
**Schematical figure to demonstrate the importance of measuring within host pathogen burden when estimating tolerance.** The panels show pathogen burden and performance profiles for two individuals differing in resistance (panels **A**), but having the same tolerance (as indicated by the slope of the solid lines in panels **B**). Here the performance trait growth rate *y* was assumed to depend linearly on pathogen burden *PB*, i.e., *y*(*t*) = *y*0(*t*) − *bPB(t*), where *y0* refers to growth rate corresponding to *PB* = 0 and *b* is the tolerance slope. For ease of illustration *y0* was assumed to differ between the individuals. The slopes of the dashed lines in panels **(B)** refer to the estimated tolerance when ignoring within host pathogen burden. panels **(C)** and **(D)** show the resulting growth rate and body weight time profiles, respectively. The dashed lines in panels **(D)** refer to the body weight profiles of both individuals in the absence of pathogen challenge. Information on how the data were generated can be found in the Appendix.

To illustrate this, consider the example illustrated in Figure 1A for two individuals exposed to the same environmental pathogen burden (or challenge dose). The individuals are assumed to differ in their resistance to the pathogen in question (Figure 1A) and (for ease of illustration) have different constant growth rates in the absence of pathogen challenge, but have the same tolerance (i.e., same reduction in growth rate with increasing pathogen burden, Figure 1B). Due to differences in resistance, the pathogen replicates at different rates within both hosts, and as a consequence the susceptible individual experiences a greater reduction in growth rate and thus also in body weight over time than the resistant individual (Figures 1C,D). Thus, comparison of performance profiles alone (Figure 1D) may reveal differences in resilience, but does not provide information on tolerance. Taking pathogen burden into account is crucial for avoiding confounding effects between resistance and tolerance. Moreover, only by considering pathogen burden explicitly can we answer the crucial question of how performance would be affected by changes in pathogen challenge (e.g., caused by epidemic outbreaks or by genetic selection for improved host resistance or tolerance).

#### The need to use within-host pathogen burden rather than environmental pathogen burden or challenge dose when quantifying tolerance

Having established that pathogen burden needs to be taken into account when measuring tolerance, the next question is how to quantify it. Given that the study of tolerance originates from ecology, where tolerance analyses follow the methodology of reaction norms (Via and Lande, 1985; Simms, 2000), i.e., the pattern of phenotypes produced by a given genotype under different environmental conditions, it may seem natural to consider pathogen burden as an environmental rather than a host characteristic.

The definition of pathogen burden as an environmental characteristic may be attractive from a practical point of view. For instance, tolerance could be obtained as the slope of performance measured in a breeding nucleus with generally low pathogen burden compared with performance in a more pathogenic commercial environment, using estimates of environmental pathogen burden in either environment. Similarly, immunologists who think of tolerance as a dose response curve (Ayres and Schneider, 2012), may define pathogen burden by the challenge dose in an infection experiment (e.g., Lefèvre et al., 2011). Both types of definitions (i.e., environmental pathogen burden or inoculation dose) thus assume that the independent variable pathogen burden is the same for all individuals and constant over time. Although attractive for practical reasons, using environmental burden or inoculation dose could however lead to biased estimates of individual tolerance due to confounding effects between resistance and tolerance. This is illustrated in Figure 1 for two individuals having the same tolerance, but differing in resistance. Although both individuals are initially challenged with the same pathogen burden, within host pathogen burden will eventually differ due to differences in host resistance (Figure 1A). At any given time post infection, the susceptible host will have greater loss in performance than the resistant host due to greater within host pathogen burden. If these differences in pathogen burden are not taken into consideration, and within host burden was replaced by a constant environmental or challenge burden in the performance *vs*. pathogen burden plot, the resulting tolerance slope would be affected. In the illustrated example (Figure 1B), the slope of the susceptible individual would become much steeper than the slope of the resistant individual, suggesting differences in tolerance despite both individuals having equal tolerance. This simple example demonstrates that quantifying tolerance requires measuring individual within-host pathogen load rather than environmental burden or challenge dose in order to avoid confounding effects between resistance and tolerance and to obtain thus unbiased tolerance estimates.

### The need to account for individual variation in performance in the absence of pathogen challenge when quantifying tolerance of a group

The definition of tolerance as a slope stipulates that multiple measurements of performance related to different levels of pathogen burden are required. This requirement has led several researchers to conclude that tolerance can only be determined at the level of groups of individuals (e.g., family, breed, or line) (Mauricio et al., 1997; Råberg et al., 2007, 2009). In fact, to the best of our knowledge, all quantitative genetic analyses of tolerance to date have specified tolerance at the level of the group rather than the individual (McIntyre and Amend, 1978; Simms and Triplett, 1994; Mauricio et al., 1997; Pilson, 2000; Kover and Schaal, 2002; Råberg et al., 2007; Blanchet et al., 2010; Lefèvre et al., 2011). In these analyses the group specific tolerance estimate is usually obtained by regressing the performance of individual group members against their respective pathogen burden recorded at a specific point in time.

**Figure 2:**
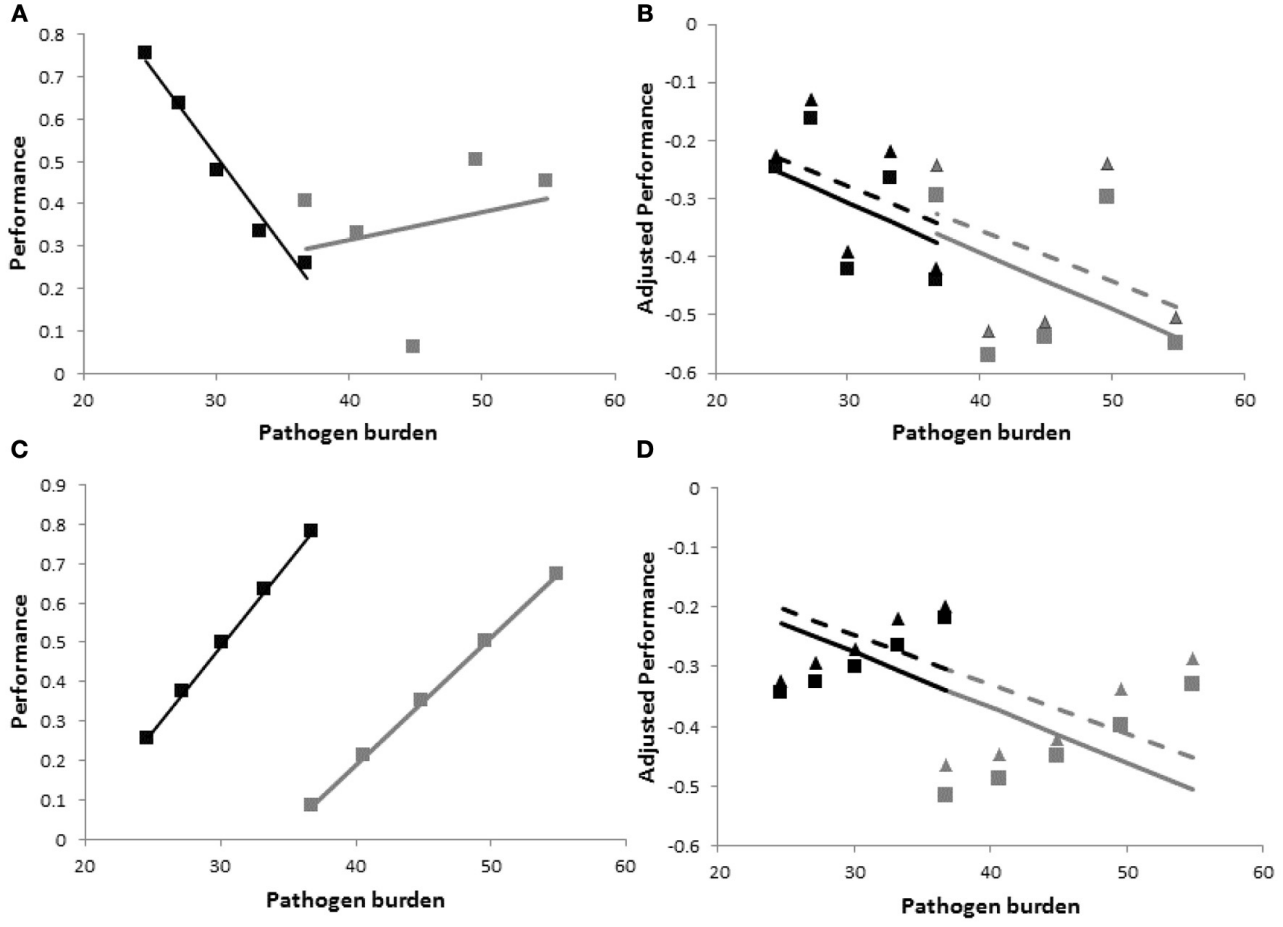
**Schematic figure to illustrate the importance of accounting for host variation in vigor, and the impact of changes in pathogen virulence and of correlation between resistance, vigor, and tolerance on resulting tolerance estimates.** The panels show hypothetical performance vs. pathogen burden plots for estimating tolerance on a family level with individual variation in resistance, vigor and tolerance. Black and gray symbols refer to individual performance vs. pathogen burden measurements at an arbitrary time point from members of two families consisting of five individuals each. Black and gray lines are the corresponding regression lines whose slope values provide the family specific tolerance estimates. The families differ in average resistance, but have the same average vigor and tolerance, and the same trait within family variances and co-variances. Top {(**A** and **B**)} and bottom {(**C** and **D**)} panels refer to zero and strong correlations between resistance, vigor and tolerance, respectively. Panels on the left {(**A** and **C**)} show actual (non-adjusted) performance vs. pathogen burden and panels on the right {(**B** and **D**)} show performance adjusted to account for individual variation in vigor *y*_*ij,adj*_ = *y*_*ij*_ − *y*0_*ij*_. Only the right hand panels (i.e., using adjusted performance) provide accurate tolerance estimates. The triangles and stippled lines in panels **(B)** and **(D)** include host-induced changes in pathogen virulence in the expressions for tolerance (i.e., *v*_*ij*_ >0 in Equation 2). These increase within family variation in performance, but do not affect resulting tolerance estimates. Information on how the data were generated can be found in the Appendix.

However, even in the case of a simple linear relationship between host performance *y* and pathogen burden *PB* for individual *i* of group *j* (as it is assumed in the majority of studies), i.e.,

(1) $$y_{ij}=y0_{ij}+b_{ij}PB_{ij}$$

there are three sources of variation between individual group members: (i) resistance, represented by heterogeneous values for *PB*_ij_, (ii) tolerance, represented by heterogeneity in the slopes *b*_ij_, and (iii) vigor, i.e., individual performance in the absence of pathogen challenge, represented by heterogeneity in the intercepts *y*0_*ij*_ (Stowe et al., 2000). Moreover, the three traits may be correlated, representing for example, trade-offs between resistance, tolerance and vigor (Mauricio et al., 1997; Agrawal et al., 1999; Pilson, 2000; Doeschl-Wilson et al., 2008). As illustrated in Figure 2, within group variation and co-variation between these traits can have a profound impact on the performance vs. pathogen burden relationship, and thus on group specific tolerance estimates. The figure depicts performance vs. within host pathogen burden for two families consisting of five individuals each. For ease of illustration it was assumed that families have the same average tolerance ($\bar{b}=-0.01$) and the same average vigor, but differ in average resistance. It was assumed that there is (the same) within family variation in all three traits, i.e., resistance (*PB*), vigor (*y0*), and tolerance (*b*). The difference between the top and bottom panels of Figure 2 is that traits are either independent (Figures 2A,B) or highly correlated (Figures 2C,D).

Figures 2A,C show that simply regressing performance against pathogen burden would lead to poor estimates of family tolerance. Not only do the regression slopes differ substantially between both families, but in some cases family tolerance slope estimates are even positive suggesting over-compensation (*b* > 0) rather than incomplete tolerance (*b* < 0). The accuracy of family tolerance estimates improves substantially after adjusting performance for individual variation in vigor (i.e., using *y*_*ij,adj*_ = *y*_*ij*_ − *y*0_*ij*_ or including *y*0_*ij*_ as a covariate in the regression analysis), as shown in Figures 2B,D.

The results of this simple simulation would thus suggest that estimating group tolerance not only requires information of individual pathogen burden and performance post infection, but also information about individual vigor. A recent simulation study has demonstrated that accounting for individual variation in vigor is not only necessary for obtaining reliable phenotypic tolerance estimates, but also for obtaining unbiased estimates of genetic parameters associated with this trait (Kause, 2011).

### The influence of host-induced change in pathogen virulence on the tolerance phenotype of a group

One important aspect that has been ignored in the definitions and approaches outlined so far is that the impact of the pathogens on host performance (here defined as pathogen virulence) may change over the time course of infection. As outlined by Ayres and Schneider (2012) changes in such pathogen virulence are likely to arise from interactions between host immune response and the pathogen, and this makes host tolerance and pathogen virulence practically inseparable. Indeed, a host may be considered to be defined as tolerant merely because it reduces pathogen virulence over time without altering the pathogen burden *per se*. For this reason it has been proposed to consider host-induced change in pathogen virulence as a tolerance effect (Little et al., 2010; Ayres and Schneider, 2012). But how does this additional component of tolerance affect the phenotypic tolerance estimates of a group of individuals?

Host-induced changes in pathogen virulence may be represented by extending the original model (1) relating the performance of an individual *i* from family *j* at time *t* to its pathogen burden as follows:

(2) $$y_{ij}(t)=y0_{ij}(t)+k_{ij}(t)PB_{ij}(t)$$

where *k*_*ij*_(*t*) is the time-dependent tolerance slope equal to *b*_*ij*_ − *v*_*ij*_ (*t*), where *b*_*ij*_ refers to tolerance in the original sense (i.e., change in performance due to change in pathogen burden) and *v*_*ij*_ (*t*) refers to the rate at which the pathogen's virulence changes over time. Thus, negative *b*_*ij*_ corresponds to incomplete tolerance and negative *v*_*ij*_ (*t*) corresponds to a reduction in the impact of pathogen burden on performance over time. If *v*_*ij*_(*t*) = 0, i.e., pathogen virulence does not change throughout the time-course of infection, Equation (2) reduces to (1). As illustrated by the triangle symbols in Figures 2B,D for *v*_*ij*_(*t*) = *v*_*ij*_ × *t* with constant rates *v*_*ij*_, changes in pathogen virulence alters host performance measured at a specific point in time without affecting the corresponding pathogen burden, and thus affects the resulting regression slopes derived from the scatter plots. This justifies statistically the inclusion of host-induced change in pathogen virulence as a component of tolerance.

It is noteworthy that the additional tolerance component introduces a further source of within family variation (and co-variation with other parameters), increasing thus the risk of errors in estimating tolerance slopes and the need for more samples to achieve statistical significance. In our simple illustrative example consisting of families with five individuals, the corresponding regression slopes were statistically significantly different (*p* < 0.05) when within family variation in pathogen virulence was added, although the average tolerance [i.e., average values for parameters *b* and *v* in equation (2)] was the same for both families. Note also that it is not possible to separate the two tolerance components (one affecting performance as a result of changing pathogen burden and one affecting the impact of a unit of pathogens on performance) when estimating group tolerance from the scatter plots. It is thus concluded that it is important to take into account that group tolerance estimates not only comprise changes in performance directly caused by changes in pathogen burden, but also by potential changes in the impact of the pathogens on host performance over time, and that both components may give rise to substantial within and between group variation.

### The influence of individual variation in the onset of infection on the tolerance phenotype of a group

In all examples shown thus far it was assumed that all individuals become infected at the same time, and that measurements are taken at the same time post-infection. Whilst these conditions can be met in artificial challenge experiments (e.g., Råberg et al., 2007; Lefèvre et al., 2011), they are unlikely to hold in natural populations where the infection spreads naturally between individuals (e.g., Blanchet et al., 2010). Can reliable estimates of group tolerance be also obtained in the case of natural transmission dynamics based on samples collected at fixed time points? Two phenomena may interfere with the estimation: firstly, not all individuals of the population may have become infected, and thus not all individuals may express tolerance. Secondly, individuals are likely to vary in exposure and consequently become infected at different times, and the onset of infection is usually unknown. The first phenomenon is likely to affect the number of samples required to achieve statistical significance. To understand the impact of the second phenomenon on group tolerance, imagine for example, two individuals with the same resistance and tolerance. If infected at the same time, the individuals would produce the same point on the performance vs. pathogen burden plot. However, the individuals may have drastically different within host pathogen burden if they became infected at different times. Without knowing the onset of infection for both individuals, it is impossible to discern whether differences in within host pathogen burden reflect differences in host resistance or differences in exposure to infection. This may introduce complications for disentangling host resistance from tolerance and produce biased tolerance phenotypes (see section “Specifying Pathogen Burden”). Similarly, both individuals may have similar pathogen burden at the time of measurement, but due to different exposures, one individual is at the early stage of infection (e.g., when pathogen burden rises in Figure 1A), whereas the other individual is already in the process of recovery (e.g., when pathogen burden declines in Figure 1A). Furthermore, if the host immune response alters pathogen virulence over the time course of infection, the individual who is at the early infection stage is likely to have a greater performance measure than the recovering individual who has been infected for longer. In this case, differences in performance rather than in pathogen burden would produce artificial differences in the tolerance slopes.

**Figure 3:**
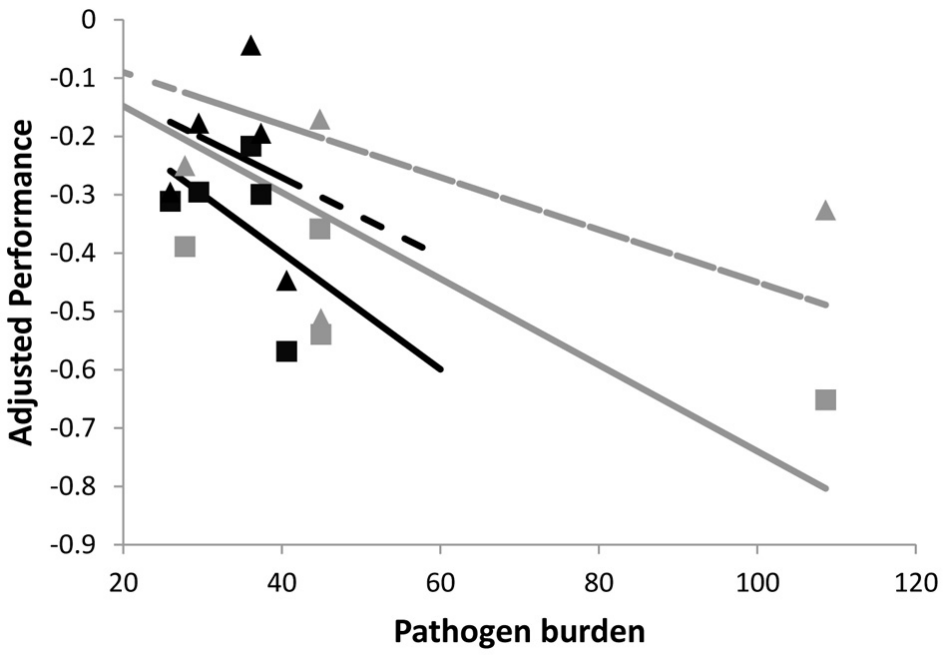
**Schematic figure to illustrate the importance of measuring pathogen burden and performance at the same time point post-infection.** The panels shows simulated performance vs. pathogen burden plots for the same individuals of the two families whose plots are shown in Figure 2B (also solid lines corresponding to no change in pathogen virulence). Families differ in average resistance but have the same average tolerance. Here, pathogen burden and performance records refer to different time points post infection, mimicking natural field conditions where individuals get infected at different times. This introduces error in the family specific tolerance estimates, as reflected by the different slopes of the corresponding (black and gray) regression lines. For further explanation see caption of Figure 2.

To further illustrate the impact of different exposure times on group tolerance estimates, Figure 3 shows cross-sectional samples of performance and pathogen burden for the same individuals of two families as simulated in Figure 2B (i.e., same average tolerance). However, in Figure 3, individuals were assumed to vary in their time of infection. This was represented by choosing the time of infection of each individual at random within a 5 day period. As a result, the corresponding family specific regression lines were no longer parallel, thus erroneously implying that one family is more tolerant than the other. In summary, individual variation in exposure blurs the distinction between resistance and tolerance and is likely to introduce bias in the phenotypic estimates of group tolerance.

## Discussion and implications

Reliable phenotypes are paramount for estimating genetic variation of traits of interest and for predicting breeding values for artificial selection. Improving tolerance of farm animals to infectious disease appears a desirable breeding goal for several reasons. However, apart from few studies investigating evidence for genetic variation in model species (e.g., Corby-Harris et al., 2007; Råberg et al., 2007; Ayres and Schneider, 2008) or wild populations (e.g., Blanchet et al., 2010; Lefèvre et al., 2011), essential knowledge of the existence and degree of genetic variation in tolerance of livestock is still lacking. We came to the conclusion that this gap of understanding may be largely due to difficulties in estimating tolerance phenotypes.

Most publications address the issue of “how to measure tolerance” either from a conceptual (e.g., Simms, 2000; Råberg et al., 2009; Ayres and Schneider, 2012) or an empirical perspective (e.g., Simms and Triplett, 1994; Mauricio et al., 1997; Råberg et al., 2007; Blanchet et al., 2010; Lefèvre et al., 2011). Conceptual studies are valuable for introducing the methodology (e.g., that tolerance can be considered as the slope when plotting performance vs. pathogen burden). However, they may not always reveal how these concepts can be implemented in practice. For example, tolerance as a concept is typically introduced at the level of individuals, but in practice it has only been estimated at the level of a group of individuals. Empirical studies, on the other hand may provide quantitative estimates of tolerance, but do not provide the necessary insight into the potential bias in these estimates as developed above. Tolerance estimates could be influenced by a variety of factors, including the time(s) at which measurements are obtained, within family variation in resistance, tolerance and vigor, and co-variation in these traits as discussed above. It is difficult to determine the impact of these factors empirically. For this reason, we combined here a qualitative literature review with some simple simulations that allow a systematic and quantitative investigation of the effects of various individual factors and their interactions on resulting tolerance estimates.

Our study emphasizes that, in comparison to other traits targeted for genetic improvement in farm animals, estimating tolerance to infectious pathogens is more complicated as it requires multiple measurements per individual. This is even the case if tolerance is defined at a group level, and contrasts with, for example, estimates of resistance that can be obtained by measuring pathogen load at a relevant point in time. For instance, in order to avoid confounding effects between host resistance and tolerance, it is critical to measure not only the performance of individuals challenged with pathogens, but also their individual within host pathogen burden in a way that it accurately reflects host resistance. Also, in order to avoid bias in the tolerance slope estimates, it is essential to record individual host performance not only when individuals are infected, but also in a non-infected state or when exposed to a different level of pathogen challenge.

We are not the first to point out that measurements of within host pathogen burdens are critical for estimating group tolerance (e.g., Simms and Triplett, 1994; Råberg et al., 2009; Ayres and Schneider, 2012; Kause and Ødegård, 2012). Indeed, most empirical evidence for genetic variation in tolerance in plants and animals to date is based on analysis of covariance, where a significant F-test for family by pathogen burden interactions implies genetic variation in tolerance. There is however ambiguity in how and when within host pathogen burden should be measured. Previous studies have used (i) pathogen (e.g., macro parasite) levels at a particular time post-infection (e.g., Simms and Triplett, 1994; Mauricio et al., 1997; Pilson, 2000), (ii) peak pathogen burden (e.g., Råberg et al., 2007), (iii) the area of the pathogen curve over the time course of infection (e.g., Rowland et al., this issue), (iv) inoculation dose (e.g., Lefèvre et al., 2011), or (v) pathogen burden of individuals infected at different time points, measured at a fixed sampling time or after death (e.g., Blanchet et al., 2010; Lefèvre et al., 2011). Our study would suggest that options (i–iii) are the least likely to introduce bias in the resulting tolerance estimates, as they do not confound resistance and tolerance effects and do not simultaneously consider individuals that differ in their infection states. In particular, option (iii) would provide estimates of host resistance and tolerance that refer to the whole time period of infection rather than to a single point in time. However, this would require repeated measures of pathogen burden and host performance over time for every individual. Controlling the time at which records are collected may not always be feasible in practice; in particular if diagnostic tests for living animals do not exist for the infection under consideration. Also, available diagnostic tests may only provide crude estimates or proxies of actual pathogen burden and thus host resistance (e.g., PCR or ELISA test providing information of whether the animal has been infected or not). Further studies would be warranted to determine how inaccuracies in pathogen burden influence the resulting tolerance estimates.

Previous studies have demonstrated that ignoring individual variation in vigor can affect inferences about host evolution (Little et al., 2010), and introduce bias in estimates of genetic variance of tolerance when vigor and resistance are correlated (Kause, 2011). Our simulations show that serious bias in the tolerance slope estimates (and therefore probably also in the estimates of genetic variance in tolerance) can occur if individual variation in host vigor is not properly accounted for. As an individual cannot be simultaneously infected and not infected, these multiple measurements on an individual would need to be obtained prior (to measure vigor) and post (to measure tolerance) challenge. This may not be difficult to achieve, particularly if challenge tests are performed. However, as discussed in our companion paper (Doeschl-Wilson et al., 2012), the time delay between successive measures may introduce the risk that factors other than pathogen burden contribute to changes in host performance leading thus to biased tolerance slope estimates. This problem is easily prevented by choosing a performance trait that is zero for all animals in the absence of pathogen challenge, such as for example infection-induced weight loss (Råberg et al., 2007) or infection-induced mortality (e.g., Corby-Harris et al., 2007; Ayres and Schneider, 2008; Blanchet et al., 2010). Note however, that not all performance traits relevant in livestock production satisfy this criterion as they are rarely in a steady state. Hence, care needs to be taken in the statistical analysis to account for factors influencing temporal changes in performance not related to pathogen challenge.

For ease of illustration we assumed a linear relationship between pathogen burden and performance in our simulations. In reality, pathogen burden may have a non-linear effect on performance. In particular, it is quite likely that pathogen burden needs to exceed a certain threshold level within the host, before impacting noticeably on performance (Sandberg et al., 2006). Also, variation in pathogen virulence between hosts may cause a more complex relationship between host performance and pathogen burden in the scatter plots that cannot be easily linearized. Our conclusions should also hold in the case of such non-linear relationships, although adaptations in the quantification of tolerance would need to be made because the slope will no longer be constant over the entire pathogen burden range. Two approaches for dealing with such non-linear relationships are presented in the literature. The first approach restricts the definition of tolerance to a range of pathogen burden values over which the slope is approximately constant [termed “range tolerance” by Little et al. (2010)]. This would imply that in order to compare tolerance of different groups of individuals, the groups need to overlap in their levels of pathogen burden. Otherwise, they may be equally tolerant but their data may refer to different sections of the performance vs. pathogen burden curve and conclusions obtained will be wrong (see e.g., Råberg et al., 2009). The second approach is to replace the slope of the regression of performance against pathogen burden with the area of performance under the pathogen burden curve, after standardizing to account for variation in vigor (Pilson, 2000).

Accurate phenotypic measures only constitute the first step toward predicting breeding values for artificial selection or for identifying loci affecting the trait under consideration. The accuracy of these genetic parameter estimates will not only depend on the quality of resistance and performance measures, but also on family size, genetic, and phenotypic correlations between this trait and resistance and vigor, and on the underlying genetic architecture (Kause, 2011). Perhaps the most limiting factor in genetic improvement of host tolerance is the fact that tolerance as a trait is a property of an individual, yet according to current methods it can only be quantified at a group level. In current breeding programs, selection across families is used as an alternative to individual selection when traits cannot be measured on selection candidates (e.g., meat quality and disease resistance traits). However, the genetic progress that can be achieved by artificial selection is limited when within family variation is ignored. Genome-wide evaluations are considered highly beneficial in such cases where individual phenotypes are difficult to obtain in practice, as they provide a means to use both between and within family variation (Sonesson and Meuwissen, 2009; Villanueva et al., 2011). But although the need of measuring the trait of interest can be avoided for some generations, individual measures are still needed for estimating SNP effects. Thus for genetic improvement programs, individual tolerance phenotypes would be highly desirable.

In conclusion, estimating tolerance phenotypes for a group of related individuals constitutes an important first step toward improving the tolerance of livestock to infectious diseases. In order to obtain unbiased estimates of group tolerance, accurate measures of within host pathogen burden and performance of individual animals, associated with different levels of pathogen burden (e.g., non-infected and infected) are needed. In order to avoid confounding effects between differences in individual resistance and environmental exposure when estimating group tolerance, individual measures of pathogen burden and host performance would need to be obtained at the same time point post pathogen exposure for all individuals. This makes estimating tolerance from field data extremely challenging.

It should be noted, that many of the issues raised here for tolerance also arise when improving host genetic resistance to infectious disease through selection (Bishop et al., 2012). In particular, as outlined by Bishop and Woolliams (2010) individual differences in pathogen exposure, appropriate timing of measurement and poor test diagnostics all contribute to potential bias in genetic parameter estimates for host resistance to infectious pathogens. Nevertheless, selection for improved host resistance has been notably successful for a variety of diseases, including nematode infections in sheep, IPN in salmon, mastitis in dairy cattle and *E. coli* infections in pigs. Although natural selection for tolerance appears to have been successful in several animal and plant species, it remains to be shown if similar success can be achieved through artificial selection. This paper contributes toward this endeavor by outlining the kind of measurements needed to make progress in this direction.

## Conflict of interest statement

The authors declare that the research was conducted in the absence of any commercial or financial relationships that could be construed as a potential conflict of interest.

## Appendix

### Supplementary information on generation of data for figures 1 and 2, respectively

The data for Figures 1 and 2 were generated using the following equations for pathogen burden (*PB*_*ij*_) of individual *i* in family *j* and performance (*y*_*ij*_):

$$\begin{array}{l} PB_{ij}(t)=100t^{2}exp\left(-c_{ij}t\right)\\ y_{ij}(t)=y0_{ij}(t)-k_{ij}(t)PB_{ij}(t) \end{array}$$

where *k*_*ij*_(*t*) = *b*_*ij*_ − *v*_*ij*_*t* refers to the tolerance slope, and *y*0_*ij*_(*t*) refers to performance at time t in the absence of pathogen challenge.

Parameter values for individuals 1 and 2 in Figure 1 were *c*_1_ = 0.8 and *c*_2_ = 0.8, *v*_i_ = 0 for *i* = 1, 2 and *y*0_1_ = 1 and *y*0_2_ = 0.5, respectively.

Parameter values for individuals in Figure 2 were

$$\begin{array}{c} c_{i1}=\left\{0.6,0.7,0.8,0.9,1.0\right\},\;c_{i2}=\left\{1.0,1.1,1.2,1.3,1.4\right\},\\ y0_{ij}\in \left\{0.6,0.7,0.8,0.9,1.0\right\},\;b_{ij}=\{0.006,0.008,0.01,0.012,0.014\}, \end{array}$$

and *v*_*ij*_ = 0 (squared symbols and solid lines) or *v*_*ij*_ ε {0.0006, 0.0008, 0.001, 0.0012, 0.0014} for triangles and stippled lines. For Figures 1A,B, individual parameter values were chosen at random, whereas for Figures 1C,D, individual parameter values were ordered to generate correlation between resistance, tolerance and vigor. Plots were generated based on predicted pathogen burden and performance at time *t* = 1.
